# Taking their wellbeing into their own hands: Self-educated and peer-recommended techniques used by women with breast cancer to improve sexual functioning during treatment and in survivorship

**DOI:** 10.1371/journal.pone.0293298

**Published:** 2023-11-15

**Authors:** Christiana von Hippel, Kate E. Dibble, Shoshana M. Rosenberg, Melissa Bollman-Jenkins, Marisa Weiss, Ann H. Partridge

**Affiliations:** 1 Department of Medical Oncology, Dana-Farber Cancer Institute, Boston, MA, United States of America; 2 Department of Social and Behavioral Sciences, Harvard T.H. Chan School of Public Health, Boston, MA, United States of America; 3 Department of Medicine, Harvard Medical School, Boston, MA, United States of America; 4 Breastcancer.org, Ardmore, PA, United States of America; Lamar University, UNITED STATES

## Abstract

**Objective:**

Coping with sexual dysfunction during and after breast cancer treatment is a persistent challenge for many women, even with clinician-offered standard sexual rehabilitative therapies (i.e., lubricants, counseling). This study sought to explore how women with breast cancer supplement clinician recommendations with self-discovered and peer-recommended techniques for improving sexual functioning and provide insight into how well they work.

**Methods:**

Adult women with stage I-IV breast cancer were recruited to participate in a one-time online survey via Breastcancer.org. Thematic analysis identified emergent domains and themes focused on techniques for improving sexual function during and after treatment. Frequencies were calculated to quantify technique sources and perceived efficacy levels.

**Results:**

Of 501 women responding to the survey, mean age was 53 years (range 30–79). Overall, 34.7% reported using a technique they discovered themselves or that was recommended by someone other than a clinician to improve sexual functioning. Four main themes regarding techniques included: 1) pain reduction, 2) intimacy and relationship enhancement, 3) desire and arousal enhancement, and 4) emotional coping. Most women discovered coping techniques without the help of clinicians, and 45.7% of women rated their techniques as moderately or more effective when used in addition to or instead of standard techniques offered by clinicians.

**Conclusions:**

Our study provides insight into how women with breast cancer successfully cope with sexual dysfunction symptoms during and after treatment. To fully understand and share patients’ innovative techniques for coping with these symptoms, clinicians should foster open discussion about the potential for dysfuction and treatment for these symptoms, as well as avenues of peer-supported discussion to promote coping self-education and discovery.

## Introduction

Sexual dysfunction, which encompasses decreased sexual desire and arousal, insufficient vaginal lubrication, and difficulty reaching orgasm, is a common sequela of breast cancer treatment, including chemotherapy and endocrine therapy [1–3]. Among breast cancer survivors, as many as 75% of those treated experience some form of sexual dysfunction [4, 5]. Conjointly, experiencing negative body image, decreased perceived sexual attractiveness to partners, and reduced self-esteem often in response to surgical modalities to treat the disease, also may increase sexual dysfunction among this population [6, 7]. Experiencing dysfunction can impede survivors’ relationships with sexual partners, inflate burden relating to stress, anxiety, and physical health [8–10], and greatly diminish survivors’ quality of life [3, 11, 12]. Persistent personal and interpersonal distress associated with sexual dysfunction remains a key factor in the clinical diagnosis of female sexual dysfunction, a medical condition characterized by persistent issues in sexual functioning [8], especially relevant to breast cancer survivor populations.

Currently recommended interventions offered to women with breast cancer have shown only moderate effectiveness in rehabilitating sexual functioning after breast cancer treatment. Rehabilitative therapies include estrogen-based tablets, vaginal rings, and creams to reduce vaginal dryness and dyspareunia, vaginal moisturizers and lubricants to hydrate vaginal tissue and ease discomfort during sexual activity [13–15], and individual or couples-based psychoeducation and sex therapy to heal intimacy issues and promote coping [16–20]. These provider-designed, therapeutic techniques are either contraindicated (as is the case of vaginal estrogens for many patients) or have not advanced enough to help the 20–30% of women with breast cancer who experience persistent sexual dysfunction challenges through treatment and into survivorship [13, 15, 16]. Women have reported that some providers do not have the time, knowledge, or resources to educate them on sexual dysfunction or interventions to assist in lessening symptoms [21] while others have been uncomfortable communicating detailed sexual symptoms and potential techniques to alleviate dysfunction [22]. Thus, the integration and value of patient-reported data into medical approaches and recommendations remains paramount in identifying complementary methods of intervention for sexual dysfunction.

Web-based forums offer an opportunity for breast cancer survivors to access peer support, including around sexual health issues. One of the internet’s largest and most active health communities is hosted by Breastcancer.org. In 2021, Breastcancer.org drew 25 million unique users worldwide, providing education and support to breast cancer survivors. Approximately 230,000 of these users regularly exchange personal information, advice and support within the Breastcancer.org community forum which discuss sexual health matters among other issues important to their quality of life [23]. However, these messages often do not fully describe the development processes and results associated with different coping strategies.

To address this gap, in partnership with Breastcancer.org, we designed a mixed-methods study 1) to explore, in narrative detail, how women with breast cancer utilize (in conjunction with, or without, clinician recommendations) self-educated and peer-recommended techniques for improving sexual functioning, and 2) to quantify the burden of sexual dysfunction among these women. We aimed to describe these techniques, who developed them, and how well women perceive them to lessen sexual dysfunction symptoms. We anticipated that within the Breastcancer.org community, some women report that they managed to improve their sexual functioning during and after treatment by actively discovering helpful coping strategies, and sharing what was successful to help others. Describing a fuller landscape of the coping methods used by breast cancer survivors to improve their sexual functioning during and after treatment can enable evaluation of specific self-discovered and peer-recommended coping methods as complements to provider-designed, therapeutic techniques commonly recommended to ameliorate sexual dysfunction symptoms.

## Materials and methods

### Study population & recruitment

Participants were recruited from the Breastcancer.org online forum community. Eligibility criteria included being ≥18 years and diagnosed with stage I-IV breast cancer. Eligibility was determined through an online, self-screening process via REDCap [24, 25] based on gender self-identification, date of birth, and disease stage. Men with breast cancer, women with breast cancer under age 18 at the time of survey completion, and women diagnosed with in situ disease were not eligible to complete the survey. Participation was not limited to survivors experiencing sexual symptoms within a specific timeframe following diagnosis as there is no clear consensus in the literature concerning when sexual dysfunction are thought to resolve in survivorship.

Recruitment was conducted through advertisements posted by the community moderators on the Breastcancer.org homepage and within relevant community forums such as the highly active “Sex and Relationship Matters” forum. The survey was open for participation between February and March 2018. Participants meeting eligibility criteria read the informational consent webpage and clicked “Continue/Proceed” to indicate passive consent. The survey was anonymous and the authors did not have access to information that could identify individual participants. This study was determined to be exempt from ethical review by the Institutional Review Board at the Dana-Farber Cancer Institute/Harvard Cancer Center (IRB#17–333).

### Mixed-methods survey protocol

A series of open- and closed-ended questions was developed and pre-tested with patient advocates regarding how women have attempted to cope with sexual dysfunction symptoms during and after breast cancer treatment (S1 File). Participants were asked whether they had ever discussed any concerns about how breast cancer treatment could affect their sex life with clinicians (“yes”, “no”, “not applicable”) and why. If participants indicated potential or current sexual dysfunction symptoms, women were asked to share what advice their clinicians had provided on this topic. Participants were also asked to describe any techniques they used to improve sexual functioning during and after breast cancer treatment, beyond those recommended by clinicians. If participants had not utilized any techniques beyond those recommended by clinicians, they were asked to describe why they had not done so. Participants who had used techniques beyond those recommended by a clinician were asked to report the developer of the techniques used (e.g., themselves, a peer, a partner).

Participants were then asked to rate the efficacy of their self-developed and/or peer-recommended techniques for managing the effects of breast cancer on their sexual wellbeing on a five-point Likert scale (“not effective at all” to “extremely effective”). The Female Sexual Function Index (FSFI) was utilized to determine the level of female sexual dysfunction symptoms to assess six domains (desire, arousal, lubrication, orgasm, satisfaction, and pain) [26, 27]. The FSFI is a 19-item Likert scale with response formats ranging from 0 or 1 (more negative) to 5 (more positive) with a range of 2.0 to 36.0 with higher scores indicating better functioning [28]. This index has excellent internal consistency of the total score (Cronbach α = 0.94) and domains (Cronbach α range, 0.85–0.94). A score of ≤26.0 has been validated as a clinical cutoff to identify the threshold for symptoms of potential dysfunction [28]. Finally, participants used a list of solution-sharing intentions (e.g., sharing it with others with breast cancer, healthcare providers, online peers, have not shared but intend to do so, do not intend to share) to report any efforts they had made to share their techniques with others (S1 File).

### Data analysis

Open-ended responses were analyzed using the standard procedures of qualitative thematic text analysis by investigator C.v.H [29–31]. C.v.H independently coded participant open-ended responses with the goal of identifying similar meanings and patterns within and across responses. Upon initial review, initial codes were developed. Participants’ descriptions regarding advice and/or techniques received from clinicians, those that were self-educated, or those that were peer-recommended were manually coded and analyzed for emergent themes. Emergent themes were defined, named, and categorized into titled domains and subsequent themes based on strength and frequency. Exemplar quotes were extracted for each theme within each domain.

Descriptive statistics, including means, medians, and frequency distributions, were used to assess participant characteristics in addition to the source and perceived efficacy level of participants’ coping techniques and solution sharing activities. The FSFI domains and total score were calculated and used for descriptive purposes only and were not indicative of diagnosing female sexual dysfunction within this sample. Participant characteristics included self-reported sociodemographic, clinical, and sexual activity status information. Statistical analyses were conducted using StataMP version 15.1 [32].

## Results

### Participant characteristics

A total of 501 participants completed the online survey an average of 10 years from their breast cancer diagnosis (range = <1–43 years) and were a median age of 53.0 at survey completion (range = 30–79 years) (Table 1). The majority of participants were early stage at diagnosis (73%), with most women diagnosed with stage I (*n* = 175, 35.0%) or II (*n* = 188, 38.0%) disease. Most reported being sexually active at the time of survey completion (*n* = 353, 70.4%), partnered (*n* = 324, 64.6%), heterosexual (*n* = 345, 68.8%), and identified as non-Hispanic white (*n* = 358, 71.4%). Approximately one quarter of participants did not provide information about their partner status (*n* = 142, 28.5%), sexual orientation (*n* = 141, 28.3%), or race/ethnicity (*n* = 111, 22.5%).

**Table 1:** Characteristics of the sample (N = 501).

Characteristic	*n* (%)
*Breast cancer stage at diagnosis*	
I	175 (35.0)
II	188 (38.0)
III	84 (17.0)
IV	54 (11.0)
*Sexual activity status*	
Currently sexually active	353 (70.4)
Sexually active in the past	143 (28.5)
Never sexually active	4 (0.7)
Missing	1 (0.4)
*Partner status*	
Partnered	324 (64.6)
Not partnered	35 (6.9)
Missing	142 (28.5)
*Sexual orientation*	
Heterosexual identified	345 (68.8)
Non-heterosexual identified	15 (2.9)
Missing	141 (28.3)
*Ethnicity*	
Hispanic/Latina	18 (3.5)
Not Hispanic/Latina	364 (72.6)
Missing	119 (23.9)
*Race*	
American Indian or Alaskan Native	6 (1.1)
Asian	8 (1.5)
Black, Haitian, or African American	18 (3.5)
Native Hawaiian or other Pacific Islander	0 (0.0)
White	358 (71.4)
Missing	111 (22.5)
	**Median (Range)**
Age at survey (years)	53 (30–79)
Time from breast cancer diagnosis (years)	10 (<1–43)

#### Qualitative emergent domains & themes in coping methods

**Fig 1:**
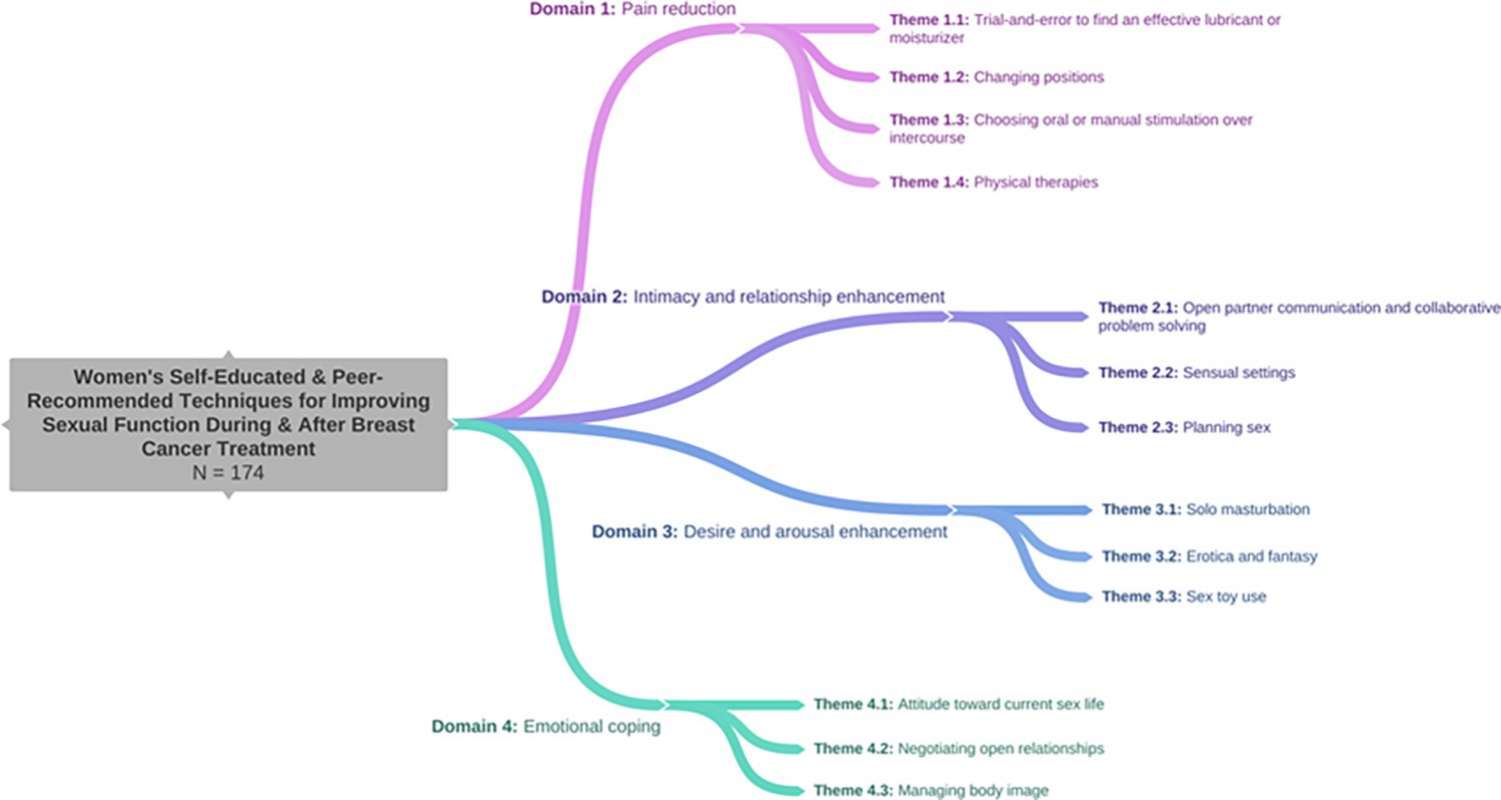
Emergent themes characterizing women’s self-educated and peer-recommended techniques for improving sexual function during and after breast cancer treatment within four domains. Created with Coggle (Coggle.it).

Of the 501 respondents, 35% (*n* = 174) reported using a self-educated technique or one recommended by someone other than a clinician to manage challenges in their sexual lives during and/or after breast cancer treatment. Of these women’s qualitative responses, four overarching domains emerged describing non-clinician recommended women’s coping techniques to improve sexual functioning. See Table 2 for exemplar participant quotes and Fig 1 for emergent domains and themes.

**Table 2:** Emergent themes characterizing women’s self-educated and peer-recommended techniques for improving sexual function during and after breast cancer treatment within four domains.

Overall Domain	Theme	Examples within Domain and Theme
**1. Pain Reduction**	**1.1.** Trial-and-error to find an effective lubricant or moisturizer	“Using coconut oil. Then freezing it in spheres using something like a melon ball maker. They can then be inserted into the vagina to get more lubrication. It should be noted that this helps but is not a fix at all.” “Discussing with fellow survivors what worked for them, as far as type of lubricants, etc. This is not the kind of thing that doctors discuss.” “Started using coconut oil as a personal lubricant–cheaper and has benefits of supposed antibacterial and anti-fungal properties.” “I received some good advice from other women on Breastcancer.org. I felt better because knew then that my symptoms were common. They gave me the idea of freezing coconut oil and using it during sex to make it less painful and it did work.” “Trying many different lubricants. Focusing on the vagina being properly cared for outside of just for sex purposes (meaning, keeping it moisturized all the time, not just right before sex).” “…a specific lubricant was suggested. The first few half dozen that I tried burned either during or after sex. I use Just Like Me a product that was introduced to me at a party for females only bedroom enhancement.”
	**1.2.** Changing positions	“We changed sides of the bed because I was more comfortable when sleeping on my side to have my right side (non-mastectomy) on the bed. Also a more comfortable position for my breast to be during foreplay.”
	**1.3.** Choosing oral or manual stimulation over intercourse	“My partner and I changed from primarily intercourse to primarily nonintercourse. This was mentioned in online forums. Oral sex or manual is less painful.” “Masturbation and have tried anal sex, but not pleasurable for me, I do it more for my husband”
	**1.4.** Physical therapies	“Occasionally a personal vibrator helps but if I do my kegel exercises properly and regularly, that helps quite a bit as well.”
**2. Intimacy and Relationship Enhancement**	**2.1.** Open partner communication and collaborative problem solving	“Talking much more with my partner, explaining what works and doesn’t work for me, asking for subtle changes in technique, looking for empathy in dealing with overwhelming fatigue at times. Also, as a result of greater openness, being able to give more to my partner, which produce a virtuous cycle and makes me feel better sexually.” “We find that depending on the cycle sometimes it’s easier than others and we try to ensure we maximize those times” “My partner is very understanding. He knows that my energy level is low so he is more physically active and I am more passive. I expect that when I feel better after treatment ends it will return to more 50/50.” “My fiancé and I always kept an open dialogue and he was very patient as well.”
	**2.2.** Sensual settings	“Open dialogue, candles, massage which made me more relaxed. My fiancé assured me I was still sexy.”
	**2.3.** Planning sex	“We plan or prepare ahead for intimacy so I can get ready mentally as well as physically.” “Daytime sex is better when I have more energy. Nighttime sex is not possible due to fatigue”
**3. Desire and Arousal Enhancement**	**3.1.** Solo masturbation	“I have tried more masturbation which has helped with my libido…” “self stimulation a couple times/week to keep the blood flow in that area” “I bought a lubricant and use it before sex. I also manually stimulate myself for a lengthy period before engaging in sexual intercourse.” “I have no partner, so masturbation is it. Use a moveable shower head most times.”
	**3.2.** Erotica and fantasy	“I find that I need more mental stimulation than I did before so suggestive texting with my husband has increased my desire.” “reading or listening to erotica,” “watching a sexy video,” “more use of imagination during intercourse”
	**3.3.** Sex toy use	“I never used one before cancer, but I have a vibrator called Entice Juliette, which is used externally. I don’t use it with my partner, just by myself.” “Use of a vibrator with a long handle so the reach is easier”
**4. Emotional Coping**	**4.1.** Attitude toward current sex life	1. “I had a mental ’ use it or lose it’ attitude and made it a priority to have sex. I have it more often than before breast cancer.” **VS.** “Acceptance that intimacy shared with my spouse with Intercourse would no longer be part of my life.”
	**4.2.** Negotiating open relationships	“Basically getting a pinch hitter for me or ignoring when he went out for a little love. . .sucks horribly but I can’t it’s too painful” “We have talked a lot about it. . .particularly my low to no libido. Along with increased fatigue (post treatment and with menopause) desire is nonexistent. There have even been discussions about opening up the marriage so that my partner can continue to fulfill their sexual needs. Thus far we have tried to work with what we have. Our sex lives have been reduced to a max of once per month (at most) from 2–3 times per week.”
	**4.3.** Managing body image	“I also myself still wore sexy lingerie that covered my scars so I could still feel and be more sexual.” “My partner understands my discomfort with my body image and understands I tire quickly. So we have lots of kisses and foreplay, I wear a loose, pretty, short nighty or top so I don’t feel exposed.” “With positive reinforcement about my reconstruction and making me feel like I am still attractive. Losing my breast (which used to bring me sexual pleasure) was a big loss for me.”

### Domain 1: Pain reduction

#### Theme 1.1: Trial-and-error to find an effective lubricant or moisturizer

Where for many it had not been necessary prior to breast cancer treatment, the majority of women who identified using a self-technique to manage challenges in their sexual lives started routinely using lubricant (e.g., estradiol, Replens, Scream Cream, vitamin E, coconut oil) to combat treatment-related vaginal dryness and reduce pain with penetration. Many have described “try[ing] one product after another after another…” to find an effective lubricant or moisturizer, or in numerous cases, a combination of both. Motivations for trying multiple products included ineffectiveness of some, irritation from ingredients, cost differences between products and product types, and ease of use (e.g., suppositories inserted every few days v. gels applied multiple times per sexual encounter). Coconut oil was the lubricant most commonly described as an effective solution for dryness, and though typically used as a salve at room temperature, some women experimented with freezing and inserting it into the vagina for prolonged relief. Additional non-traditional suppositories used by several women included vitamin E capsules, as they were described as a cost-effective alternative to non-estrogenic moisturizing creams.

#### Theme 1.2: Changing positions

Many women reported trying different sex positions than they had used before breast cancer. For instance, women who had unilateral mastectomy described lying on their unaffected side during foreplay and intercourse to reduce pressure on the surgical site.

#### Theme 1.3: Choosing oral or manual stimulation over intercourse

To maintain an active sexual life without pain from penetration, some women engaged in oral sex as their primary/most frequent form of sexual activity. Others chose mutual masturbation or anal sex with male partners as an alternative to vaginal intercourse.

#### Theme 1.4: Physical therapies

Women most frequently referenced using two forms of physical therapy for the vagina: Kegel exercises that strengthen the pelvic floor and improve ease of orgasm; and dilators used to stretch the canal to gradually increase penetration tolerance. In these cases, therapies were recommended by peer online and internet resources. Less commonly referenced physical therapies included vacuum pumps to stimulate blood flow to the clitoris and yoga to promote whole-body relaxation and flexibility.

### Domain 2: Intimacy enhancement

#### Theme 2.1: Open partner communication and collaborative problem-solving

Many women described the importance of openly communicating with their partners about what feels good sexually, what does not, and how these preferences may change over the course of treatment and into survivorship. Some referred to the coping process as collaborative, with the engagement of each partner a process of trial-and-error to cultivate a satisfying sexual life during and after breast cancer treatment. Women noted certain qualities of their partners as facilitators of this collaborative, sexual problem-solving process included patience, encouragement, and willingness to try new things.

#### Theme 2.2: Sensual settings

A reported facilitator of intimacy was romantic atmosphere, involving both the physical setting (e.g., candles, music) created by the woman and her partner in addition to her partner’s verbal affirmations of the woman’s desirability.

#### Theme 2.3: Planning sex

Some women described the need to prepare mentally and physically for sex, which involved advanced planning with their partner for the type and timing of their next sexual encounter. Some of these plans revolved around when they had more time to stimulate desire and arousal in various ways (e.g., solo masturbation, wearing lingerie, reading erotica) as a precursor to sex with a partner. Others cited fatigue as a motivation for planning to have sex during times of day or of their menstrual cycle when they typically felt most energetic.

### Domain 3: Desire & arousal enhancement

#### Theme 3.1: Solo masturbation

Solo masturbation, mentioned by partnered women, was described as something concsciously practiced in order to maintain vaginal blood flow and increase desire for partner sex. One woman described masturbating on her own for a short time as a helpful preparatory routine prior to intercourse with her partner. Some unpartnered women cited masturbation as their primary form of sexual activity/gratification. Methods of solo masturbation included stimulation with hands, sex toys, and removable showerheads.

#### Theme 3.2: Erotica and fantasy

Some women described needing more mental stimulation to inspire desire and arousal than prior to their breast cancer treatment. Methods included having more frequent sexual fantasies, watching pornographic videos, reading erotic stories, and sending erotic text or email messages to partners to stimulate desire and arousal.

#### Theme 3.3: Sex toy use.

Many women described integrating sex toys into partnered sex. The only toys referred to by name were vibrators. Vaginal vibrators were described by women as helpful for self-pleasuring as well as stretching the vagina to regain elasticity. Therefore, in some women’s experience, vibrator use overlapped with the physical therapy Theme 1.4 functioning as a dilator.

### Domain 4: Emotional coping

#### Theme 4.1: Changing attitude toward current sex life

Women who described mindset as a factor in coping with sexual dysfunction noted one of two primary attitudes: one of persistence or one of acceptance. A subset of women adopting an attitude of persistence made it a point to remember how much they enjoyed sex with their partner(s) prior to breast cancer as motivation to prioritize their effort to regain that sexual satisfaction despite challenges of pain and discouragement. Others, however, framed their persistence with partner sex despite low desire, motivated by a “use it or lose it” mindset, hoping that their desire would improve with time or would help prevent vaginal atrophy. These women had sex with their partners at the same or a higher frequency than they had pre-treatment.

Women adopting an attitude of acceptance, on the other hand, found grieving the loss of the ability to have intercourse after breast cancer and then moving on to be an effective emotional coping strategy. Some women with this mindset no longer attempted to find methods to facilitate intercourse but still engaged in other forms of sexual activity. Few accepted the loss of their sex life in its entirety and no longer sought any form of sexual activity.

Unpartnered women were a minority within the current sample. The majority of unpartnered women did not speak to their physicians about the impact breast cancer treatment may have on their sex lives, but many noted that it was never mentioned or did not feel comfortable discussing it. Despite this, many noted that their main priority was not sex, but rather, “focused primarily on treatment and survival”.

#### Theme 4.2: Negotiating open relationships

Among partnered women who adopted an attitude of acceptance toward the loss of the sex life they once enjoyed prior to breast cancer (Theme 4.1), a few considered opening the relationship sexually. They framed this decision as an emotionally painful, but viable option, for satisifying their partner’s need for sexual intimacy while also keeping their loving, long-term relationship intact.

#### Theme 4.3: Managing body image

Women used a number of clothing-related strategies to improve their perception of their own desirability after physical changes from treatment, especially post-mastectomy. Women specifically sought lingerie that would hide surgical scars and still make them feel sexy. Others remained half-clothed, not specifically in lingerie, during sex to improve their psychological comfort with their partners seeing their body. Some described buying or making their own soft prosthetics to wear inside their bra during sex for both comfort and aesthetics.

### Quantitative outcomes

#### Sexual functioning challenges & associated clinician advice

A total of 234 women (46.7%) reported being very or extremely satisfied with their sex lives prior to breast cancer treatment, and 43.9% (*n* = 220) reported having a very much worsened sex life post-diagnosis. A subset of 130 participants (25.9%) reported discussing concerns about how breast cancer treatments could affect their sex life with their clinicians (i.e., oncologists, cancer nurses, gynecologists). Two of the most common concerns reported were vaginal dryness (*n* = 81, 62.3%) and pain with penetration (*n* = 79, 60.7%) associated with chemotherapy and endocrine therapy. Subsequent clinician recommendations to address these reported symptoms were the use of lubricants and moisturizers (*n* = 47, 36.1%) such as Scream Cream and Replens, or Premarin or lidocaine numbing creams (*n* = 7, 5.3%). Three women reported using Scream Cream, a prescription compound applied to the clitoris to increase vasodilation and sensitivity, to reach orgasm post-treatment. These women approached their clinician for a Scream Cream prescription after discovering the cream through discussion with peers on Breastcancer.org. Two of the three women described identifying the ingredients themselves so that their clinicians can write a custom compound prescription. None of the three women who reported using Scream Cream described resistance from their doctors for the request of this relatively new, peer-recommended therapy.

The majority of participants (*n* = 287, 57.2%) reported never talking to their clinician about the sexual side effects of breast cancer treatment and/or potential remedies. Potential reasons for lack of discussion included women’s unawareness that treatment could affect their sex life, lack of comfort with talking to their provider about sex, belief that sexual issues were outside of their provider’s area of expertise or interest, and women’s own exclusive focus on survival over quality of life while in treatment. Others believed the discussion of sexual concerns was not applicable, with some citing that they did not want to seek help for their loss of libido post-treatment, while 27 (5.3%) did not respond to whether they had discussed these concerns with their clinicians.

Of the total sample, 233 (46.5%) FSFI cases were analyzed. Cases were removed if participants did not complete ≥8 FSFI items (110, 21.9%) [28]. The average scores of each domain were as follows: desire (*M* = 2.57, *SD* = 1.20, range = 1.2–6.0), arousal (*M* = 2.93, *SD* = 1.48, range = 0.0–6.0), lubrication (*M* = 3.00, *SD* = 1.81, range = 0.0–6.0), orgasm (*M* = 3.13, *SD* = 1.72, range = 0.0–6.0), satisfaction (*M* = 3.49, *SD* = 1.48, range = 0.8–6.0), and pain (*M* = 3.51, *SD* = 2.00, range = 0.0–6.0). The average FSFI total score was 18.6 (*SD* = 7.44, range = 2.0–35.7), with 47 (20.1%) of the women completing the FSFI meeting the clinical cutoff for sexual dysfunction.

#### Coping techniques, perceived effectiveness & solution sharing activities

Of the 174 women who reported trying a coping technique recommended by someone other than a clinician, approximately 36.2% (*n* = 63) of women used more than one technique. For instance, one woman reported combining peer-recommended techniques of frozen coconut oil to reduce pain, wearing lingerie to cover scars, and utilizing an open partner dialogue to enhance sexual functioning. Additionally, 77 women (44.2%) reported discovering their own techniques or in conjunction with partners (*n* = 54, 31.0%, Table 3). Technique diffusion was high, however. A total of 149 women reported sharing their solutions, so that others including peers (*n* = 39, 26.1%) and clinicians (*n* = 48, 32.2%) could learn from their experience (Table 4). Of the 174 women who reported using a coping technique recommended by someone other than a clinician, 54 (31.0%) reported no intention of sharing techniques/coping methods with others at all. Of the 174 participants who rated their technique effectiveness, 45.9% (*n* = 80) reported their techniques as moderately or more effective when used in addition to or instead of standard therapies offered by clinicians, while 43.6% (*n* = 76) rated their techniques as slightly effective, and 10.9% (*n* = 19) as ineffective.

**Table 3:** Non-clinical developers of techniques used by women with breast cancer for improving sexual function (N = 174 participants).

Non-clinical developers	*n* (%) of women using techniques from each source
The woman with breast cancer herself (i.e., self-educated)	77 (44.2)
The woman with breast cancer in collaboration with her intimate partner(s)	54 (31.0)
Another person with breast cancer (i.e., peer-recommended)	37 (21.2)
A family member or friend	13 (7.4)
Someone the woman with breast cancer read about on the internet	37 (21.2)
Someone the woman with breast cancer heard about on TV or the radio	2 (1.1)
Other (not specified)	9 (5.1)

*Note*. Total exceeds 100% because many participants used techniques from more than one source and therefore, selected more than one type of developer.

**Table 4:** Solution sharing activities of women with breast cancer who used a self-educated or peer-recommended technique for improving sexual function (N = 149).

Solution sharing activities	*n* (%) of women engaged in this activity
Told other women with breast cancer	39 (26.1)
Told healthcare providers	48 (32.2)
Shared a description of coping methods on a website/blog/social network such as Breastcancer.org	32 (21.4)
Have not shared yet, but do intend to tell others	43 (28.8)
No intention to share coping methods with others	54 (36.2)

*Note*. Total exceeds 100% because many participants engaged in more than one solution sharing activity.

## Discussion

Many breast cancer survivors experience challenges in their intimate lives both during and after treatment, which may persist for an extended period of time. In this survey of an Internet-based community of women treated for breast cancer, a wide variety of effective, self-educated and peer-recommended techniques for increasing intimacy, desire, and arousal, reducing vaginal pain and coping emotionally with changes in sexual functioning after initiating breast cancer treatment were reported. In some instances, women 10 years or more from diagnosis were still actively coping with sexual dysfunction that had arisen during or shortly after breast cancer treatment, a pattern common among breast cancer survivors [33–35].

Thus, women often looked for personal coping solutions beyond those suggested by their clinicians such as those recommended by peers. There exist many barriers to doctor-patient communication, especially in relation to breast cancer treatment-related sexual dysfunction, like older age, openness, and lack of time [36, 37]. Often, patients expect oncologists to be experts in areas where they are without training or experience like managing side effects of treatment [36]. For instance, the use of Scream Cream, a compounded topical cream comprised of the natural hormone oxytocin, one bronchodilator, and two vasodilators, is more often prescribed by gynecologists and more specifically, gynecologists specializing in postmenopausal care compared to oncologists [38].

It was common to undergo a process of trial-and-error leading up to the discovery of one, or often multiple, coping techniques perceived to improve sexual functioning. The experimentation during this time was often supported by collaboration with partners. In fact, women frequently credited their partners for assisting in their innovative coping techniques, which has been noted in previous literature [37]. Therefore, we included women and their partner(s) in our definition of “self-educated” coping innovative. This definition respects couples and women with multiple partners as units of self-experimentation, important for understanding patient problem-solving in other aspects of wellbeing beyond sexual functioning that include a large interpersonal component.

The sample was homogeneous, with most patients identifying as non-Hispanic white, heterosexual, partnered, and being diagnosed with early stage breast cancer. Most women, therefore, described sexual challenges and coping mechanisms in relation to female-male partner sex, particularly strategies for improving their comfort with having and talking about intercourse. Within this context, trying new sexual techniques and evaluating their success for facilitating partner sex typically require partners’ consent and thus, invited collaboration. Previous randomized clinical trials have recommended the use of couples’ and/or sex therapy that fostered open communication in conjunction with medical advice and prescription creams, lotions, and ointments to improve sexual functioning [37, 39, 40]. Once a successful solution was found, women and their partners often continued using it as indicated by the present tense language used by many participants.

### Clinical implications

Consistent with previous research among women with breast and other cancers [41–43], the majority of women in the current sample did not discuss potential or current sexual impacts of breast cancer treatment with their providers. They did, however, describe coping techniques and strategies beyond provider guidance, since most women never received any advice from providers regarding sexual health or dysfunction. Clinicians should raise the topic when discussing breast cancer treatments in conjunction with their potential impact on quality of life, so that patients can be informed about what to expect and how to address any challenges if they should occur. Additionally, routinely addressing sexual functioning during follow-up care will allow clinicians to offer appropriate and timely sexual rehabilitative therapies to survivors experiencing symptoms of sexual dysfunction [42]. Some of the self-educated and peer-recommended techniques women described in the current study, namely lubricants, moisturizers, and physical therapies, are not well-known to clinicians, especially oncologists, with minimal knowledge regarding sexuality after breast cancer and its solutions, as this information is available, but not within their regular experience and training [21, 43]. This may indicate that in many cases, solutions to treatment-related symptoms of sexual dysfunction could have been offered to survivors directly, had the conversation had been addressed in the clinic. However, given that standard therapies are often insufficient to manage sexual functioning challenges during and after treatment [7, 8, 13, 15, 17, 44], clinicians should also encourage women’s safe experimentation with their own techniques for reducing symptoms, either supported by clinicians or in the context of a safe moderated community like Breastcancer.org [45]. There are additional online resources such as the American Cancer Society or Susan G. Komen [33, 46] for breast cancer patients and survivors.

Clinicians can encourage and support patients’ safe experimentation with coping techniques, by inquiring not just about survivors’ experiences of sexual dysfunction symptoms, but also by sharing any *solutions* women may have attempted or found for improving sexual functioning. Although the minority of our sample had discussed sexual dysfunction symptoms with clinicians, many survivors who tried a self-educated or peer-recommended technique to lessen these symptoms expressed openness to sharing solutions they found with providers. In fact, this subgroup’s dissemination of solutions to clinicians was more common in the current study compared to comparable studies of other patient populations actively coping with chronic diseases [47]. The high proportion of women who made efforts to share their coping techniques or strategies with clinicians and others were likely facilitated by the nature of the Breastcancer.org community as an engaged group of communicative breast cancer survivors already acquainted to trading personal and sensitive advice online. Therefore, the initiation of sexual functioning-related discussions by providers may be well-received by many breast cancer patients and survivors. It is also possible, for instance, that breast cancer survivors, especially long-term survivors, are over-attributing their sexual dysfunction symptoms to breast cancer treatment [48]. Normalizing the cause of sexual dysfunction beyond breast cancer (e.g., aging, lifestyle behaviors) may lead to a greater peace of mind and broader solutions.

Facilitating open discussions of sexual challenges and patient-led problem-solving strategies may vary by clinician. Clinically, standardized female sexual dysfunction measures may be utilized, to screen for the sexual dysfsunction symptoms [28]. Using these measures may act as a pre-screening tool to open conversation. When open conversation is supported, symptom-related treatment options can be discussed openly. In the current study, a small number of women discussed peer-recommended prescription-only Scream Cream with their clinician for increasing sexual desire, demonstrating promise for discussions such as these. Clinicians without the knowledge and/or experience of, or those who do not feel comfortable discussing, sexual challenges with breast cancer patients and survivors can also engage other members of their team (e.g., nurses, navigators, sexual counselors) to initiate and facilitate a discussion on sexuality with their patients. It is also helpful to refer women to other providers with relevant experience (e.g., gynecologists, urologists) or to platforms like Breastcancer.org [45] for peer-to-peer support and information exchange on the topic of coping with sexual dysfunction.

### Study limitations

The generalizability of our findings is limited due to the homogeneity of the sample. Participants were predominantly non-Hispanic white, partnered, and heterosexual women, which reflects Breastcancer.org user demographics. Additionally, most women reported early stage disease, whose sexual function may be very different from those with metastatic disease, who are more likely to be under continuous treatment and experience pain, fatigue, stress, and other symptoms. Similarly, the average age of the sample was 53 years, making it possible that symptoms of sexual dysfunction (i.e., vaginal dryness, dyspareunia, changes in self-image or -esteem, etc.) may be due to the onset of menopause in some women. It is also important to note that there are subgroups of breast cancer survivors from different sociocultural backgrounds that will provide unique experiences and information regarding sexual dysfunction and self-educated or peer-recommended coping techniques that differ greatly from those discussed in this paper. Future research should focus on recruiting women from diverse racial, ethnic, and sexual minority groups, in addition to those from low socioeconomic positions to fully incorporate sexual dysfunction care access and participant-derived methods of relief. Analytically, only one qualitative coder analyzed the data for the current study, precluding additional chance for discussions regarding domains and themes that an additional coder might have contributed. An additional limitation involves some women choosing to provide more complete descriptions of their coping techniques than others. It is possible that having to type open-ended responses to survey prompts eliminated the richness or dearth of data that was analyzed in the current study. Initial or follow-up interviews in support of typed responses could be conducted to provide a more detailed narrative may be beneficial.

### Future research

It remains imperative to vet the safety, quality, and generalizability of women’s more novel techniques for coping with sexual challenges both during and after breast cancer treatment. The findings of the current study were reported by a homogeneous sample of non-Hispanic white heterosexual women with breast cancer. It remains increasingly important to identify symptoms of sexual dysfunction among more diverse samples of breast cancer survivors. Future research would benefit by recruiting from smaller, more targeted support groups or online forums in tandem with larger organizations like Breastcancer.org. National organizations like the African American Breast Cancer Alliance (AABCA), the Sisters Network, Inc., and the Latino Cancer Institute for instance, have members that can offer perspectives different from those discussed here. Additionally, current age should be considered when discussing issues with sexual functioning, as the onset of menopause may be contributing to the experience and/or severity of such symptoms. There exists a gap in the literature regarding this online community and similar communities of women with other cancers who are open to discussing their experience of sexual dysfunction for which they have self-educated or tried peer-recommended solutions in place of, or in addition to, clinician recommendations. Evaluation-focused research like the current study will enable clinicians, researchers, and other subject area experts to identify promising techniques and assess their effectiveness as a complement to standard, clinician-developed therapies. Recent research has begun to highlight the use of vaginal laser therapy (also called the MonaLisa Touch Laser, as noted by some participants) for improving sexual dysfunction symptoms among menopausal breast cancer survivors has been published [49–52]. It is important to discern that the Food and Drug Administration has not cleared or approved this treatment for female sexual dysfunction or geritourinary symptoms of menopause due to breast cancer treatment [53]. Future research should, however, ask participants about the type of clinician they are seeking, or have sought, sexual health solutions from, what was recommended to them, and how successful these techniques or products were.

In the current study, coping techniques utilized by women with breast cancer for improving sexual functioning were largely behavioral strategies (e.g., partner communication techniques, modes of dress) as opposed to implementing the use of innovative sexual wellness products (e.g., homemade lubricant, modified sex toys). Given that lubricants, topical creams/gels, and/or moisturizers were the most commonly used type of coping technique, it is necessary for research to discern the most effective product(s) including the safety (including hormone-based products) and efficacy of various types and their doses. These findings expand the literature on patient-educated innovation that has, to this point, primarily captured product- and prescription-focused innovations [15, 38]. Thus, quality of life-improving product innovation has been observed at a high rate among patients living with rare and chronic diseases, especially among male patients [47, 54]. One potential reason for our success with capturing women’s behavioral innovations in this study is the strategy-oriented framing of our request for women to describe all of the self-educated and peer-recommended *techniques* they have used to manage challenges in their sexual lives both during and after breast cancer treatment. The trust built between Breastcancer.org and breast cancer patients/survivors could have prompted greater participation in an online survey focusing on the particularly private topic of sexuality. Future research using this technique-based framing of inquiry may have success with identifying behavioral and product innovations in both woman-dominant and mixed-sex cancer patient communities.

## Conclusions

Our findings begin to provide insight into how women with breast cancer successfully meet their needs for coping support outside the context of clinical treatment. Efforts to understand and diffuse patients’ innovative techniques for coping with persistent sexual dysfunction symptoms post-treatment have promise for benefitting the quality of life of the broader population of women with breast and other cancers.

## Supporting information

S1 File Participant survey including sociodemographic and clinical cancer information. (PDF)

S1 Data Deidentified dataset. (CSV)
