# Supplementary material for: Taking their wellbeing into their own hands: Self-educated and peer-recommended techniques used by women with breast cancer to improve sexual functioning during treatment and in survivorship
Source: PLoS One. 2023 Nov 15;18(11):e0293298. doi: 10.1371/journal.pone.0293298 (PMC10650983; doi:10.1371/journal.pone.0293298)
Supplement: S1 File — (PDF) [file pone.0293298.s001.pdf]

Supplement File 1. Participant Questionnaire

Unless otherwise directed, please choose only one answer for each question.

Section 1. Experience of sexual life and coping after breast cancer treatment
We will get started with some questions about your experience of sexual wellness since your breast cancer diagnosis and treatment. Many women experience loss of desire for sex during and after breast cancer treatment. Sometimes the hormonal changes that many women experience can cause sex to become painful and therefore not enjoyable while others do not find that treatment impacts their sex life much. Every woman's experience is different. Please choose one answer and take your time.

1. Are you sexually active? Sexual activity includes sex with partner(s), masturbation, intercourse, and other sexual activities with partner(s).
   - ☐ Yes, currently
   - ☐ Yes, in the past, but not now
   - ☐ No, never

2. Prior to having breast cancer, how satisfied were you with your sex life?
   - ☐ Extremely satisfied
   - ☐ Very satisfied
   - ☐ Moderately satisfied
   - ☐ Somewhat satisfied
   - ☐ Not at all satisfied
   - ☐ N/A

3. How has having breast cancer affected your sexual satisfaction when you have sex?
   - ☐ Very much improved
   - ☐ Improved
   - ☐ No effect
   - ☐ Worsened
   - ☐ Very much worsened
   - ☐ N/A

4. What, if any, differences have you experienced in your sex life since undergoing breast cancer treatment?

   <u>Open-ended question followed by blank text box</u>

Now we are hoping to learn about how you got information about the potential effects of breast cancer treatment on sexual wellbeing starting with what you learned from your doctor. Not all doctors bring it up and not all women know to ask about this topic. We'd like to hear about what this was like for you.

5. Did you and your doctor or healthcare team ever discuss concerns about how breast cancer could affect your sex life?

- ☐ Yes - Please give us a sense of what the doctor/healthcare team suggested you do to manage sexual symptoms and whether you tried it. Please answer in the space that pops up below this question.

  Open-ended question followed by blank text box

- ☐ No - If not, what prevented you from talking to your doctor/healthcare team about how breast cancer could affect your sex life? Please answer in the space that pops up below this question.

  Open-ended question followed by blank text box

- ☐ N/A – I did not speak to my doctor about sexual health issues because they were not relevant to me.

Sometimes, women with breast cancer may need ways to cope with or manage their symptoms ***beyond the advice they get from their doctor.*** *For instance, they may ask another survivor what worked for her or find an idea online and give that strategy a try. Other times women and their partners come up with innovative new ideas for how to cope because what's out there is not quite sufficient to meet their needs. You might think of this as being resourceful or adapting your normal routine to make daily life and sex easier after cancer. Coping methods that are* ***not formal medical treatments or a doctor's advice*** *can include using:*

- *Strategies for making life and sex with the disease easier. These could be big or small changes you've made to your behavior or mentality that came out of the need to be resourceful in daily life, such as tips and tricks for: better managing pain or discomfort, boosting sexual desire, communicating openly with partner(s), trying different sex positions to find what feels best for you post-treatment, and many others.*
- *DIY ("Do-It-Yourself") treatments or therapies, such as non-prescription supplements, yoga, vaginal steaming to stimulate blood flow, meditation, etc.*
  Health-related products such as sex toys you've modified to work better for you, homemade lubricant, customized post-surgical clothing, homemade breast prosthetics, etc. These are not prescription products.

6. Are you actively coping with the sexual symptoms of your breast cancer treatment?
   - ☐ Yes, I have been for MORE than 6 months.
   - ☐ Yes, I have been for LESS than 6 months.
   - ☐ No, but I intend to in the next 30 days.
   - ☐ No, but I intend to in the next 6 months.
   - ☐ No, and I do NOT intend to in the next 6 months.
   - ☐ N/A – I was not affected by sexual functioning issues

7. Have you tried a coping method that you developed yourself or someone other than a healthcare provider suggested (e.g., another breast cancer survivor, your partner(s), a friend, etc.)?
   - ☐ Yes
   - ☐ No [SKIP TO ANSWER QUESTION 9]

☐ N/A [MOVE ON TO BEGINNING OF SECTION 2]

8. What were the coping methods that you developed yourself or that someone other than a healthcare provider suggested? Please describe them.

   Open-ended question followed by blank text box
   [SKIP TO ANSWER QUESTION 10]

9. If you have not tried using a coping method other than the ones suggested by a healthcare provider to deal with sexual symptoms, what are the main reasons you didn't? Please answer in the space below.

   Open-ended question followed by blank text box
   [MOVE ON TO BEGINNING OF SECTION 2]

10. Would you consider any of your coping methods to be new ideas or strategies that are somehow unique to you?
    - ☐ Yes
    - ☐ No
    - ☐ Not sure

11. Who developed the coping method(s) you tried? (Check all that apply.)
    - ☐ I did
    - ☐ My intimate partner(s) and I came up with it/them together
    - ☐ A family member or friend
    - ☐ Another person with breast cancer
    - ☐ A doctor or other healthcare professional
    - ☐ Someone I read about on the internet
    - ☐ Someone I heard about on TV or the radio
    - ☐ Other (please specify): _________________

12. Coping in the context of sex life often involves a woman's partner(s). Have your sexual partner(s) been involved in figuring out ways to enjoy sex after your breast cancer treatment?
    - ☐ Yes
    - ☐ No
    - ☐ Not sure

13. *How* have your sexual partners (current or past) been involved in figuring out ways to enjoy sex after your breast cancer treatment?

    Open-ended question followed by blank text box

14. Overall, how effective were the coping methods you used for making it easier to manage the effects of breast cancer on your sexual wellbeing?

- Not effective at all
- Slightly effective
- Moderately effective
- Very effective
- Extremely effective

15. How did you share what you came up with/what coping methods helped you? (Please check yes or no.)
    - I told other people with breast cancer
      - Yes
      - No
    - I told healthcare providers
      - Yes
      - No
    - I shared my coping methods on a website/blog/social network such as Breastcancer.org
      - Yes
      - No
    - I have not shared yet but I do intend to tell others
      - Yes
      - No
    - I do not intend to share my coping methods
      - Yes
      - No

Section 2. Sexual Activity and Female Sexual Function Index (FSFI)
We will shift gears to a standard measure of sexual wellness that asks about your sexual feelings and responses during the ***past 4 weeks****. Please answer the following questions as honestly and clearly as possible. CHECK ONLY ONE BOX PER QUESTION. Your responses will be kept completely confidential. But if there's a question you'd rather not answer, that's fine too.*

In answering these questions the following definitions apply:

- ***Sexual activity*** *can include caressing, foreplay, masturbation and vaginal intercourse.*
- ***Sexual intercourse*** *is defined as penile penetration (entry) of the vagina.*
- ***Sexual stimulation*** *includes situations like foreplay with partner(s), self-stimulation (masturbation), or sexual fantasy.*

  Sexual desire *or* ***interest*** *is a feeling that includes wanting to have a sexual experience, feeling receptive to partners' sexual initiation, and thinking or fantasizing about having sex.*

16. Over the past 4 weeks, how would you rate your level (degree) of sexual desire or interest?
    - Very high
    - High
    - Moderate

- [ ] Low
- [ ] Very low or none at all

17. Over the past 4 weeks, how often did you feel sexual desire or interest?
    - [ ] Almost always or always
    - [ ] Most times (more than half the time)
    - [ ] Sometimes (about half the time)
    - [ ] A few times (less than half the time)
    - [ ] Almost never or never

18. Over the past 4 weeks, how frequently have you engaged in self-stimulation (masturbation)?
    - [ ] Not at all
    - [ ] Once
    - [ ] A few times this month
    - [ ] Once a week
    - [ ] Two to three times per week
    - [ ] Almost every day
    - [ ] More than once per day

19. Over the past 4 weeks, how frequently have you engaged in sexual activity with one or more partners?
    - [ ] Not at all
    - [ ] Once
    - [ ] A few times this month
    - [ ] Once a week
    - [ ] Two to three times per week
    - [ ] Almost every day
    - [ ] More than once per day

Sexual arousal *is a feeling that includes both physical and mental aspects of sexual excitement. It may include feelings of warmth or tingling in the genitals, lubrication (wetness), or muscle contractions.*

20. Over the past 4 weeks, how often did you feel sexually aroused ("turned on") during sexual activity or intercourse?
    - [ ] Almost always or always
    - [ ] Most times (more than half the time)
    - [ ] Sometimes (about half the time)
    - [ ] A few times (less than half the time)
    - [ ] Almost never or never
    - [ ] No sexual activity

21. Over the past 4 weeks, how would you rate your level of sexual arousal ("turn on") during sexual activity or intercourse?

- Very high
- High
- Moderate
- Low
- Very low
- No sexual activity

22. Over the past 4 weeks, how confident were you about becoming sexually aroused during sexual activity or intercourse?
    - Very high confidence
    - High confidence
    - Moderate confidence
    - Low confidence
    - Very low or no confidence
    - No sexual activity

23. Over the past 4 weeks, how often have you been satisfied with your arousal (excitement) during sexual activity or intercourse?
    - Almost always or always
    - Most times (more than half the time)
    - Sometimes (about half the time)
    - A few times (less than half the time)
    - Almost never or never
    - No sexual activity

24. Over the past 4 weeks, how often did you become lubricated ("wet") during sexual activity or intercourse?
    - Almost always or always
    - Most times (more than half the time)
    - Sometimes (about half the time)
    - A few times (less than half the time)
    - Almost never or never
    - No sexual activity

25. Over the past 4 weeks, how difficult was it to become lubricated ("wet") during sexual activity or intercourse?
    - Extremely difficult or impossible
    - Very difficult
    - Difficult
    - Slightly difficult
    - Not difficult
    - No sexual activity

26. Over the past 4 weeks, how often did you maintain your lubrication ("wetness") until completion of sexual activity or intercourse?

- ☐ Almost always or always
- ☐ Most times (more than half the time)
- ☐ Sometimes (about half the time)
- ☐ A few times (less than half the time)
- ☐ Almost never or never
- ☐ No sexual activity

27. Over the past 4 weeks, how difficult was it to maintain your lubrication ("wetness") until completion of sexual activity or intercourse?
    - ☐ Extremely difficult or impossible
    - ☐ Very difficult
    - ☐ Difficult
    - ☐ Slightly difficult
    - ☐ Not difficult
    - ☐ No sexual activity

28. Over the past 4 weeks, when you had sexual stimulation or intercourse, how often did you reach orgasm (climax)?
    - ☐ Almost always or always
    - ☐ Most times (more than half the time)
    - ☐ Sometimes (about half the time)
    - ☐ A few times (less than half the time)
    - ☐ Almost never or never
    - ☐ No sexual activity

29. Over the past 4 weeks, when you had sexual stimulation or intercourse, how difficult was it for you to reach orgasm (climax)?
    - ☐ Extremely difficult or impossible
    - ☐ Very difficult
    - ☐ Difficult
    - ☐ Slightly difficult
    - ☐ Not difficult
    - ☐ No sexual activity

30. Over the past 4 weeks, how satisfied were you with your ability to reach orgasm (climax) during sexual activity or intercourse?
    - ☐ Very satisfied
    - ☐ Moderately satisfied
    - ☐ About equally satisfied and dissatisfied
    - ☐ Moderately dissatisfied
    - ☐ Very dissatisfied
    - ☐ No sexual activity

31. Over the past 4 weeks, how satisfied have you been with the amount of emotional closeness during sexual activity between you and your partner(s)?
    - ☐ Very satisfied
    - ☐ Moderately satisfied
    - ☐ About equally satisfied and dissatisfied
    - ☐ Moderately dissatisfied
    - ☐ Very dissatisfied
    - ☐ No partner(s)
    - ☐ No sexual activity with my partner(s)

32. Over the past 4 weeks, how satisfied have you been with your sexual relationship with your partner(s)?
    - ☐ Very satisfied
    - ☐ Moderately satisfied
    - ☐ About equally satisfied and dissatisfied
    - ☐ Moderately dissatisfied
    - ☐ Very dissatisfied
    - ☐ No partners

33. Over the past 4 weeks, how satisfied have you been with your overall sexual life?
    - ☐ Very satisfied
    - ☐ Moderately satisfied
    - ☐ About equally satisfied and dissatisfied
    - ☐ Moderately dissatisfied
    - ☐ Very dissatisfied

34. Over the past 4 weeks, how often did you experience discomfort or pain during vaginal penetration?
    - ☐ Almost always or always
    - ☐ Most times (more than half the time)
    - ☐ Sometimes (about half the time)
    - ☐ A few times (less than half the time)
    - ☐ Almost never or never
    - ☐ Did not attempt intercourse

35. Over the past 4 weeks, how often did you experience discomfort or pain following vaginal penetration?
    - ☐ Almost always or always
    - ☐ Most times (more than half the time)
    - ☐ Sometimes (about half the time)
    - ☐ A few times (less than half the time)
    - ☐ Almost never or never
    - ☐ Did not attempt intercourse

36. Over the past 4 weeks, how would you rate your level (degree) of discomfort or pain during or following vaginal penetration?
    - ☐ Very high
    - ☐ High
    - ☐ Moderate
    - ☐ Low
    - ☐ Very low or none at all
    - ☐ Did not attempt intercourse

Section 3. Your Background and Breast Cancer Care

The next questions about your background and breast cancer care. In this section, we want to hear about you and how you're feeling these days. ***For each question, please check the one box that corresponds to your answer (unless otherwise indicated).***

37. In general, would you say your health is:
    - ☐ Excellent
    - ☐ Very good
    - ☐ Good
    - ☐ Fair
    - ☐ Poor

38. At what age were you diagnosed with breast cancer?
    Drop-down menu of years of age

39. Are you in a long-term relationship? (This includes being married, living as married, having a long-term dating partner(s), etc.)
    - ☐ Yes
    - ☐ No

40. Do you think of yourself as…
    - ☐ Heterosexual or straight
    - ☐ Homosexual, gay, or lesbian
    - ☐ Bisexual
    - ☐ Something else [Please describe: ________________________________]

41. Are you currently pregnant?
    - ☐ Yes
    - ☐ No
    - ☐ Not sure

42. Do you have any children?
    - ☐ Yes, they live at home all or part of the time
    - ☐ Yes, but they do not live at home
    - ☐ No

43. Approximately how old were you at the date of your last menstrual period? If you are still menstruating, you can select your current age.
Drop down menu of ages

44. Have you had chemotherapy?
 Yes, I am currently on chemotherapy
 Yes, I have had chemotherapy in the past
 No

45. Have you had hormonal therapy (e.g. tamoxifen or an aromatase inhibitor: anastrozole/Arimidex, letrozole/Femara, exemestane/Aromasin)?
 Yes, I am currently on hormonal therapy
 Yes, I have been on hormonal therapy in the past
 No

46. Have your ovaries ever been suppressed or removed?
 Yes, removed. Check yes, even if you also had ovarian suppression in the past.
 Yes, currently suppressed
 Yes, suppressed in the past
 No, never suppressed or removed

47. Have you had any of the following surgical treatments for breast cancer? **Please check off all types that apply.**
 Yes, mastectomy with breast reconstruction
a. Unilateral mastectomy (one breast removed)
b. Bilateral mastectomy (both breasts removed)
 Yes, mastectomy but no breast reconstruction
a. Unilateral mastectomy (one breast removed)
b. Bilateral mastectomy (both breasts removed)
 Lumpectomy (or partial mastectomy)
 Full lymph node dissection
 Sentinel lymph node biopsy
 No, I have not had surgery

48. What is your current height (in feet and inches, approximately)?
Drop down menu

49. What is your current weight (in pounds, approximately)?
Drop down menu

50. What is the highest level of education that you have completed?
 Grade school/junior high
 Some high school
 High school graduate
 Technical/vocational school

- Some college
- College graduate
- Post-college graduate

51. Do you consider yourself to be Hispanic or Latina? ***(Spanish, Hispanic, or Latina*** is a person of Mexican, Puerto Rican, Cuban, South or Central American, or other Spanish culture of origin, regardless of race.)
    - Yes
    - No

52. What race do you consider yourself to be? **Please check all that apply.**
    - **American Indian or Alaskan Native (Native Person/Aboriginal).** A person having origins in any of the original peoples of North, Central, or South America, and who maintains tribal affiliation or community attachment.
    - **Asian.** A person having origins in any of the original peoples of the Far East, Southeast Asia, or the Indian subcontinent, including for example, Cambodia, China, India, Japan, Korea, Malaysia, Pakistan, the Philippine Islands, Thailand, and Vietnam.
    - **Black, Haitian, or African American.** A person having origins in any of the black racial groups of Africa.
    - **Native Hawaiian or other Pacific Islander.** A person having origins in any of the original peoples of Hawaii, Guam, Samoa, or other Pacific Islands.
    - **White.** A person having origins in any of the original peoples of Europe, the Middle East, or North Africa.

53. Where do you live?
    Drop-down menu of country
    If US selected, menu of US states

54. What is your current employment status? **Please check off all that apply.**
    - Currently working full time
    - Currently working part time
    - Unemployed
    - Self-employed
    - Student
    - Homemaker
    - Disabled
    - Retired
    - Other (specify): _______

55. Which of these categories best describes your total combined family income for the past 12 months? This should include income (before taxes) from all sources, wages, rent from properties, Social Security, disability and/or veteran's benefits, unemployment benefits, workman's compensation, help from relatives (including child payments and alimony), and so on.

- ☐ Less than $25,000
- ☐ $25,000 - $49,999
- ☐ $50,000 - $74,999
- ☐ $75,000 - $99,999
- ☐ More than $100,000
- ☐ Don't know
- ☐ I prefer not to answer

56. How would you describe your household's financial situation right now? **Please check off only one.**
    - ☐ After paying the bills, you still have enough money for special things that you want.
    - ☐ You have enough money to pay the bills, but little spare money to buy extra or special things.
    - ☐ You have money to pay the bills, but only because you have cut back on things.
    - ☐ You are having difficulty paying the bills, no matter what you do.

57. In the past week, on how many days have you done a total of 30 minutes or more of physical activity, which was enough to raise your breathing rate. This may include sports, exercise, and brisk walking or cycling for recreation or to get to and from places, but should not include housework or physical activity that may be part of your job.
    - ☐ 0
    - ☐ 1
    - ☐ 2
    - ☐ 3
    - ☐ 4
    - ☐ 5
    - ☐ 6
    - ☐ 7

58. How many alcoholic beverages (beer, wine, mixed drinks, etc.) do you currently consume weekly?
    - ☐ None
    - ☐ Less than 1 drink a week
    - ☐ 1 to 4 drinks per week
    - ☐ 5 to 9 drinks a week
    - ☐ 10 to 19 drinks a week
    - ☐ More than 19 drinks a week

59. Have you ever smoked?
    - ☐ Yes, but only in the past
    - ☐ Yes, currently
    - ☐ No, never

60. The following questions refer to how much you have been bothered by any of the following problems **during the PAST 4 WEEKS. Choose one number on each line. If you do not have the problem, check off "not at all."**

During the past 4 weeks, I have had:	Not at all	Slightly	Moderately	Quite a bit	Extremely
a) hot flashes					
b) night sweats					
c) nausea					
d) vomiting					
e) difficulty with bladder control when laughing or crying					
f) difficulty with bladder control at other times					
g) vaginal dryness					
h) pain with intercourse					
i) general aches and pains					
j) joint pains					
k) muscle stiffness					
l) forgetfulness					
m) difficulty concentrating					
n) problems with being easily distracted					
o) weight gain					
p) unhappiness about the appearance of my body					
q) arm swelling (lymphedema)					
r) decreased range of motion in the arm on the side where I had surgery					
s) fatigue, tiredness					

61. Since you first had breast cancer, has breast cancer come back in other places in your body?
- ☐ Yes
- ☐ No
- ☐ Not sure

62. Are you living with metastatic disease?
- ☐ Yes
- ☐ No
- ☐ Not sure

63. We would like to know if you have any of the following common medical conditions in the table below, and how much they affect your day-to-day activities.

If you do not currently have the health condition, please check "no" in column A, and skip to the next problem.

If you currently have the health condition:

Please check "yes" in column A, and continue on to columns B & C.
In Column B, please indicate if you receive medications or some other type of treatment for the problem.
In column C, please indicate if the problem limits any of your activities.

	A		B		C	
**Heath Condition**	Do you have the health condition		Do you receive treatment for it?		Does it limit your activities?	
Heart disease	No	Yes→	No	Yes	No	Yes
High blood pressure	No	Yes→	No	Yes	No	Yes
Lung disease	No	Yes→	No	Yes	No	Yes
Diabetes	No	Yes→	No	Yes	No	Yes
Ulcer or stomach disease	No	Yes→	No	Yes	No	Yes
Kidney disease	No	Yes→	No	Yes	No	Yes
Liver disease	No	Yes→	No	Yes	No	Yes
Anemia or other blood disease	No	Yes→	No	Yes	No	Yes
Cancer (excluding breast)	No	Yes→	No	Yes	No	Yes
Depression	No	Yes→	No	Yes	No	Yes
Osteoarthritis, degenerative arthritis	No	Yes→	No	Yes	No	Yes
Back pain	No	Yes→	No	Yes	No	Yes
Rheumatoid arthritis	No	Yes→	No	Yes	No	Yes
Other medical problems (please specify)	No	Yes →	No	Yes	No	Yes
Other medical problems (please specify) ____________________	No	Yes→	No	Yes	No	Yes
Other medical problems (please specify) ____________________	No	Yes→	No	Yes	No	Yes
Other medical problems (please specify) ____________________	No	Yes→	No	Yes	No	Yes
Other medical problems (please specify)	No	Yes→	No	Yes	No	Yes

Click SUBMIT to save and submit your survey answers
